# Timing to restart direct oral anticoagulants after traumatic intracranial haemorrhage (RESTART tICrH): study protocol for a randomised controlled multicentre trial

**DOI:** 10.1136/bmjopen-2026-121224

**Published:** 2026-09-17

**Authors:** Ellie Edlmann, Siddarth Kannan, Laura Wright, Ben Hardwick, Michaela Brown, Matthew Gornall, Terence McLoughlin, Victoria Haunton, Yankier Pijeira Perez, Dyfrig A Hughes, Nic Vine, Helen Grantham, Truman J Milling, Deirdre A Lane, Gregory Y H Lip, Michael D Jenkinson, Catherine J McMahon

**Affiliations:** 1 Department of Neurosurgery, University of Plymouth, Plymouth, UK; 2 South West Neurosurgical Centre, University Hospitals Plymouth NHS Trust, Plymouth, UK; 3 Neurosurgery, Walton Centre for Neurology and Neurosurgery, Liverpool, UK; 4 Liverpool Clinical Trials Centre, Liverpool, UK; 5 Emergency Department, Royal Liverpool University Hospital, Liverpool, UK; 6 Faculty of Health, Plymouth University, Plymouth, UK; 7 Centre for Health Economics and Medicines Evaluation, Bangor University, Bangor, UK; 8 Public and Patient Involvement representatives, London, UK; 9 University of Texas at Austin Dell Seton Medical Center, Austin, Texas, USA; 10 Department of Cardiovascular and Metabolic Medicine, Institute of Life Course and Medical Sciences, University of Liverpool, Liverpool, UK; 11 Liverpool Centre for Cardiovascular Science, Liverpool John Moores University, Liverpool, UK; 12 Aalborg Universitet, Aalborg, Denmark; 13 University of Bialystok, Bialystok, Podlaskie Voivodeship, Poland; 14 University of Liverpool Institute of Systems Molecular and Integrative Biology, Liverpool, UK; 15 Neurosurgery, Northern Care Alliance NHS Foundation Trust, Salford, UK

**Keywords:** TRAUMA MANAGEMENT, Anticoagulation, Stroke

## Abstract

**Background:**

Head injury in older adults is increasingly common, often caused by low-impact falls. A significant proportion of these patients are taking oral anticoagulants (OACs) to prevent stroke or thromboembolism. When traumatic bleeding within the head occurs, OACs are usually stopped to reduce the risk of worsening haemorrhage; however, prolonged interruption increases the risk of stroke, systemic thromboembolism and death. There is currently no consensus on when it is safest to restart OACs. Direct oral anticoagulants (DOACs) are now widely prescribed and may offer advantages over warfarin. This trial aims to determine the safest and most effective timing to start/restart a DOAC after a traumatic intracranial haemorrhage (tICH).

**Methods:**

This is a phase III, multi-centre, randomised controlled trial. Adults with tICH who were taking an OAC before injury will be recruited across approximately 20 trauma networks in the UK. Eligible participants will be randomised to restart DOAC therapy either 1 week or 4 weeks after tICH. The primary outcome is the proportion of patients with a haemorrhagic or thrombotic event within 12 weeks of tICH. Secondary outcomes include time-to-first haemorrhagic or thrombotic event, survival, functional status, quality of life and health resource use. Follow-up will occur at 6, 12 and 26 weeks, with data analysed on an intention-to-treat basis. Health economic and qualitative sub-studies will run alongside the main trial.

**Discussion:**

This study addresses a major area of clinical uncertainty. By directly comparing 1-week versus 4-week re-initiation of OAC, this trial will provide robust evidence to guide clinical practice in a tICH population at high risk of both bleeding and thrombotic complications. Findings will inform patient counselling, acute care protocols and guideline development.

**Ethics and dissemination section:**

Ethical approval has been obtained from the Berkshire Research Ethics Committee (24/SC/0298). Written informed consent will be obtained from all participants or their legal representatives before enrolment. Findings will be disseminated through peer-reviewed publications, scientific conferences, trial registry reporting and patient-facing summaries, with the aim of informing future guidance on anticoagulation management following tICrH.

**Trial registration:**

NCT06322953.

**STRENGTHS AND LIMITATIONS OF THIS STUDY**

- Multicentre, pragmatic randomised controlled trial evaluating the optimal timing of direct oral anticoagulant resumption after traumatic intracranial haemorrhage.
- Includes clinical, functional, quality-of-life, health economic and qualitative outcomes.
- Patient and public involvement informed the study design and outcome selection.
- Open-label design may introduce bias, although it reflects routine clinical practice.
- Findings may not be generalisable to patients requiring long-term vitamin K antagonist therapy or those with mechanical heart valves.

## Introduction

 Head injury in older adults is increasingly common. In the 2017 review of trauma networks in England and Wales, a fall from standing height in an older person was the most common mechanism of major trauma and most often resulted in head injury (these data excluded isolated neck of femur and distal limb fractures).^1^ With one in six people projected to be aged ≥65 years by 2050, and around 30% of older adults falling each year, the incidence of head injury will continue to increase.^2 3^ In parallel, oral anticoagulant (OAC) use has grown because atrial fibrillation (AF)—whose prevalence rises with age—increases stroke risk about five-fold and merits OAC based on stroke risk, commonly assessed using the CHA_2_DS_2_VASc or CHA_2_DS_2_VA clinical risk scores.^4 5^

OAC prescribing in the UK has more than doubled since 2000,^6^ and 20–35% of older patients admitted with traumatic intracranial haemorrhage (tICrH) are taking an OAC at the time of injury.^7 8^ Standard practice is to stop OAC acutely to mitigate early haemorrhage expansion; however, despite the low risk of new or ongoing intracranial bleeding after 48 hours, clinicians are often reluctant to restart OAC because of rebleeding concerns. This must be balanced against the increased risk of stroke, systemic thromboembolism and death when anticoagulation is withheld.^9 10^ There is no clear consensus on optimal timing for restarting OAC after tICrH and a 2021 survey of UK neurosurgeons reported that most advise restarting OACs at 1–4 weeks following tICrH.^6^ A live poll during a virtual UK neurosurgical conference confirmed acceptability of early (1 week) compared with late (4 weeks) and this is also supported by a proposed trial design by an American study group which included 1, 2 and 4 week restart times.^7^

One recent trial assessing patients with major bleeding on anticoagulation reported re-bleeding rates of 4.3% within 30 days when excluding the first 3 days after the initial bleed.^8^ Importantly, the bleeding rate was only 4.5% among those who restarted anticoagulation within 2 weeks compared with those who did not restart within the same timeframe. Another study focusing specifically on tICrH observed a 5% readmission rate for re-bleeding within 30 days in patients who did not restart their direct oral anticoagulant (DOAC).^11^ Despite these findings, there remains a paucity of high-level data describing delayed (>72 hours) haemorrhage rates after tICrH.

OAC medications are generally divided into vitamin K antagonists (eg, warfarin) and DOACs. DOAC use has risen substantially over the past decade given the advantages of rapid starting, fixed dosing, fewer drug interactions and no routine monitoring compared with warfarin.^12 13^ Trials and meta-analyses show that DOACs are at least as effective as warfarin for stroke prevention in AF, with lower rates of intracranial haemorrhage and mortality.^14^ Similar evidence for efficacy and safety is available for venous thromboembolism.^15^

This trial will focus on patients prescribed a DOAC or warfarin for atrial fibrillation or venous thromboembolism at the time of tICrH whose OAC is stopped acutely. Patients will either restart their DOAC or, if on warfarin or another vitamin K antagonist (VKA), be offered a switch to a DOAC in line with best practice and the superior safety profile of DOACs after intracranial haemorrhage.^16^

## Methods and analysis

### Objectives

Our primary objective is to compare the clinical effectiveness (a reduction in thrombotic events outweighing any increase in haemorrhagic events) of restarting or initiating a DOAC at 1 week versus 4 weeks following tICrH. Secondary objectives are to evaluate the effect of early versus late anticoagulation restart on (1) timing of haemorrhagic/thrombotic events and survival; to better understand how events relate to the original head injury and DOAC timing; (2) functional status and health-related quality of life with measures relevant to long-term recovery in the older head injury population; and (3) qualitative data on patient and caregiver attitudes. The primary economic objective is to estimate cost-effectiveness. A summary of the study outcomes and their definitions is provided in table 1.

**Table 1:** Primary and secondary outcomes

Outcome	Definition / Measurement	Timepoint
Primary outcome		
Proportion of patients experiencing a haemorrhagic or thrombotic event following traumatic intracranial haemorrhage.		12 weeks
Event definition*	Haemorrhagic events: (i) haematoma expansion or new intracranial haemorrhage on CT/MRI; (ii) change in clinical status (altered conscious level, new or progressing neurological deficit); and (iii) cessation of anticoagulation if already restarted. Thrombotic events: venous thromboembolism, myocardial infarction, ischaemic stroke, systemic embolism or non-bleeding cardiovascular death.	—
Secondary outcomes		
Time to first haemorrhagic or thrombotic event	Time (days) from randomisation to first qualifying event.	Up to 12 weeks
Time to first haemorrhagic event	Time (days) from randomisation to first haemorrhagic event.	Up to 12 weeks
Time to first thrombotic event	Time (days) from randomisation to first thrombotic event.	Up to 12 weeks
Survival	Time from randomisation to death from any cause.	Up to 26 weeks
Functional outcomes	Simplified Modified Rankin Scale, Barthel Index, Extended Glasgow Outcome Scale and Rockwood Frailty Score.	12 and 26 weeks
Quality of life	EQ-5D-5L scores; utilities used for health-economic evaluation.	6, 12 and 26 weeks
Patient and caregiver attitudes	Semi-structured interviews exploring views on starting or restarting oral anticoagulation.	Within 60 days
Healthcare resource use	Hospital, community and social-care utilisation derived from case report forms and routine data linkage (eg, Hospital Episode Statistics).	6, 12 and 26 weeks
Economic Outcome		
Cost-effectiveness	Incremental cost per quality-adjusted life year (QALY) gained	Lifetime

* Events are determined by the clinical team caring for the patient.

### Patient and public involvement

Patients and caregivers were involved in formulating the study question and informing the study design. There was clear consensus that both preventing a stroke and re-bleeding were to be avoided which informed the decision to use a composite primary endpoint. All patients were happy to be randomised to restarting a DOAC at week 1 or week 4. There was a clear preference for remote follow-up by phone, post or email. Legal representative consent will be used in cases where the patients lack capacity to consent. Two patients with lived experience of taking DOAC are members of the trial management group and will contribute to ongoing trial oversight, participant-facing materials and interpretation of findings. Their involvement will ensure that the study remains aligned with patient priorities, supports acceptable recruitment and retention strategies and enhances the relevance and accessibility of trial outputs. Public and patient involvement (PPI) contributors will also be involved in dissemination activities, including development of lay summaries and communication of results to participants and the wider public.

### Trial design

This trial is designed as a multicentre, pragmatic, parallel-group, two-arm, superiority, randomised controlled trial (RCT), with 1:1 allocation ratio, stratified by recruitment site, injury severity (Glasgow Coma Score >12 or ≤12) and frailty assessed via the Rockwood Clinical Frailty Scale (0–4 or 5–9). The study is a Type A CTIMP (Clinical Trial of Investigational Medicinal Product): no higher than that of standard medical care. Participants will be identified and recruited across at least 20 trauma networks in the UK (https://restart-trial.org.uk). Eligible participants are adults with tICrH who were receiving oral anticoagulation as part of their routine medications prior to admission, regardless of when the last dose was taken. Full inclusion and exclusion criteria are summarised in table 2.

**Table 2:** Eligibility criteria for participation in the trial

Category	Criteria
Inclusion criteria	Informed consent obtained from participant or participant’s legal representative and ability to comply with the requirements of the trial. Adults ≥18 years with traumatic intracranial haemorrhage (tICrH) within the past 1 week who were taking an OAC prior to admission. Oral anticoagulants include any direct oral DOAC or VKA (eg, warfarin), prescribed for AF or VTE prior to admission for tICrH.
Exclusion criteria	Traumatic intracranial haemorrhage limited to chronic subdural haematoma only. Patients with a mechanical heart valve. Plan to start/restart antiplatelet therapy within 12 weeks of tICrH. Abbreviated Injury Scale other than head with a score >3. Pregnant or breast-feeding female. Participants of reproductive potential (male or female) unwilling to use a reliable means of contraception. Hypersensitivity or contraindication to DOAC as detailed in the Investigational Medicinal Product Summary of Product Characteristics (SmPC). Ongoing bleeding where it would be unsafe to restart DOAC at 1 week. Clinical reason requiring DOAC restart before 4 weeks or beyond the 12-week study period. Indication to remain on VKA rather than switching to DOAC (eg, severe renal impairment or mechanical heart valve).

AF, atrial fibrillation; DOAC, direct oral anticoagulant; OAC, oral anticoagulant; VKA, vitamin K antagonist; VTE, Venous Thromboembolism.

### Intervention and comparator

Adults with tICrH who require OAC will be randomised to re/start a DOAC either 1 week or 4 weeks after the index haemorrhage. The specific DOAC (eg, apixaban, dabigatran, edoxaban, rivaroxaban) and dose will be prescribed as part of usual care according to the patient’s prior prescription or local hospital policy. The trial does not mandate a particular agent if switching from warfarin or other VKA. Sites will verify that participants initiate DOAC at the allocated time point and ascertain whether doses have been missed at each follow-up. Participants (or a legal representative) can receive reminders to re-start by text message, email, phone or video call, as per their preference.

Discontinuation or modification of DOAC therapy is determined on clinical grounds by the local medical team; participants remain in the trial and reasons are recorded on the electronic case report form. Prespecified circumstances prompting treatment cessation include adverse events, use of prohibited medication, pregnancy or any condition that in the investigator’s judgement jeopardises participant safety.

### Qualitative sub-study

An embedded qualitative sub-study will explore patient and caregiver attitudes to recommencing a DOAC following tICrH to support understanding and communication of final study findings to both clinicians and patients and identify knowledge gaps for future research. A PPI focus group of 6–8 tICrH survivors and caregivers will be undertaken to inform the topic guide as there is currently limited qualitative data available in this patient group.^17^ Purposive sampling will be undertaken to obtain 10–20 patients and/or caregivers (of those patients lacking capacity to consent) to represent diversity in terms of site, age, gender, ethnicity, presence of a caregiver and other patient characteristics (PROGRESS+criteria).^18^ Participants who decline (n=10 patient/caregivers) will also be invited to take part to discuss reasons for declining a DOAC and to inform patient-clinician discussions regarding treatment options. Individual semi-structured interviews will be undertaken, data collection will continue until saturation is reached, prior to thematic framework analysis.^19^

### Harms and event reporting

Anticipated risks reflect the clinical trade-off inherent in restarting anticoagulation after tICrH and include recurrent or progressive intracranial haemorrhage and extracranial bleeding versus thrombotic events if anticoagulation is withheld. Safety events will be reported from consent to the last study visit (week 26 or premature discontinuation). Related adverse events will be collected for this trial; haemorrhagic or thrombotic events that constitute prespecified trial endpoints are captured as outcomes and will not be duplicated as adverse events. Thrombotic events are determined by imaging initiated by clinicians on the basis of patient symptoms and clinical suspicion as part of routine care, rather than any pre-determined screening. As these are outcome events they will be screened for at all follow-up points with the research team but do not require immediate reporting. Haemorrhagic events are also determined by imaging initiated by clinicians; however, to avoid over-reporting of minor or insignificant imaging changes, must also be accompanied by a clear clinical concern with new neurological symptoms or altered conscious level. In the case of patients who have restarted their DOAC this must also be considered significant enough that the clinician wishes to stop the DOAC. There is no central adjudication or pre-specified determinants of haemorrhagic events and the study relies on pragmatic clinician-led decision making.

All events meeting the definition of a serious adverse event must be reported to the coordinating centre within 24 hours of site awareness. All reportable adverse events will be graded for severity (mild: no interference with routine activities; moderate: interferes with routine activities; severe: impossible to perform routine activities; life-threatening; death). The following are not recorded as adverse events: non-serious events that are unrelated; medical/surgical procedures per se (record the underlying condition instead); pre-existing conditions that do not worsen; situations without an untoward medical occurrence (eg, elective cosmetic surgery); overdose without signs or symptoms (though this may still prompt safety follow-up to ensure regulatory compliance). If overdose leads to an event meeting, it will be reported accordingly and highlighted to the coordinating centre.

### Sample size

Due to the lack of data in the tICrH population alone, event rate data was calculated from a post-analysis of a trial assessing patients with a major bleed while on a DOAC (64% of whom had an intracranial haemorrhage).^20^ The reported re-bleeding rate was around 4%, thrombotic event rate 9.7%, and composite event rate (including death) of 13.6% at 30 days. Just under 1/3 of patients had re-started their anti-thrombotic by 30 days and no thrombotic events occurred after re-starting DOACs. When using a landmark analysis to compare patients who re-started their DOAC versus not restarted, a 14-day cut-off had a HR of 0.384 for composite events. This suggests a 60% reduction in events if restarting by 2 weeks, and likely to be greater for restarting at 1 week. The haemorrhagic events they were similar between those who had restarted (4%) and not (4.4%).

We have extrapolated these data to our cohort with a combined event rate of 12% (3% haemorrhagic and 9% thrombotic events within 12 weeks of tIcH) in the 4-week restart group and a 6% absolute reduction in the 1-week restart group (3% and 3%), assuming a significant reduction of thrombotic events without an increase in haemorrhagic events. Using a group sequential design with a single interim analysis after 50% of patients have reached 12 week follow-up or experienced an event. Sample size calculations were undertaken in SAS software version 9.4 with SAS/STAT package 14.3. The O’Brien-Fleming spending function, based on standardised Z-statistics, was used to determine the efficacy and futility boundaries (non-binding). For an overall two-sided 5% significance level and 90% power, 487 participants are required per group. To allow for 10% loss to follow-up, the total was increased to 542 per group (1084 in total).

### Randomisation

Randomisation lists were produced by an independent statistician at the Liverpool Clinical Trials Centre (LCTC), who is otherwise not involved in the trial. The trial uses restricted randomisation with variable block lengths, a 1:1 allocation ratio, and stratification by site, injury severity and frailty. Participants will be randomised via a secure web-based randomisation system centrally controlled by the LCTC once eligibility criteria have been confirmed, informed consent has been obtained, and pre-randomisation baseline assessments have been completed. The randomisation system will be established, maintained and monitored independently of the trial statistician and other trial staff. Personnel responsible for enrolling participants will not have access to the allocation sequence, and assignment to intervention groups will be performed by independent staff separate from those involved in enrolment, ensuring that allocation remains unbiased.

### Blinding

This is an open-label, pragmatic, parallel-group RCT. No blinding will be implemented for participants, treating clinicians, site research staff and outcome assessors. The trial management group including the lead statistician (MB) and health economist (YPP and DAH) will remain blinded to treatment allocation until trial completion and database lock to minimise bias during the study. The trial statistician (MG) will be unblinded once the statistical analysis plan has been finalised in order to produce the Independent Data and Safety Monitoring Committee (IDSMC) reports.

### Data collection and management

**Figure 1:**
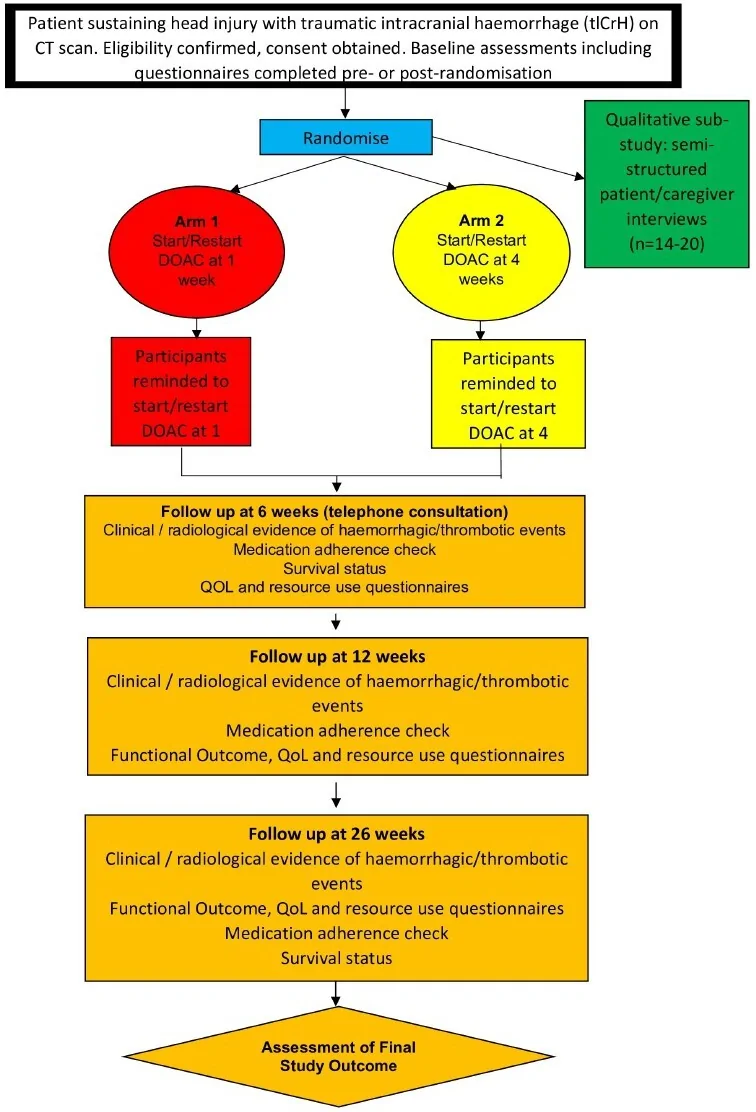
Trial Flowchart.

Trial outcomes will be assessed using validated instruments, including the Simplified Modified Rankin Scale, Barthel Index, Extended Glasgow Outcome Scale, Rockwood Frailty Score and EQ-5D-5L (Health Related Quality of Life score) (online supplementary table 1).^21–25^ Clinicians will be involved in the radiological and clinical evaluation of haemorrhagic and thrombotic events. Baseline data collection will include demographic characteristics, medical history, vascular risk factors, functional assessments and neuroimaging scored using the Liverpool Head Injury Tomography Score.^26^ Outcomes will be collected at predefined time points (baseline, 6, 12 and 26 weeks) using standardised electronic case report forms and questionnaires (figure 1).^27^ All data will be entered by authorised site staff into a secure, web-based trial database, with programmed validation checks and central monitoring to ensure accuracy and completeness. To enhance participant retention and ensure complete follow-up, participants will be contacted after randomisation by text, email or telephone to encourage adherence to the assigned intervention and scheduled assessments. In cases of missed visits, sites will attempt to contact the participant and reschedule the missed visit within 2 weeks. Before a participant is deemed to be lost to follow-up, site research staff will make every effort to regain contact with the participant and document in patient clinical records. Any participants lost to follow-up will be recorded in the trial database.

### Statistical methods

All analyses will follow the principle of intention-to-treat, as far as practically possible, and this approach will be applied to both the primary and secondary outcomes. Missing data will be actively monitored throughout the trial. Strategies will be implemented to minimise missingness, and the reasons for missing data will be systematically recorded to inform the appropriate handling of missing data in the analysis.

A single interim analysis will be conducted once 50% of the total participants have been recruited and followed up to 12 weeks, or once an event has been observed, whichever occurs first (to allow primary outcome to be analysed). Non-binding stopping boundaries will be applied using the O’Brien–Fleming spending function based on standardised Z-statistics, with efficacy and futility boundaries set at 2.963 and 0.345, respectively.

The primary outcome: proportion of patients experiencing a haemorrhagic or thrombotic event following tICrH, will be analysed using logistic regression adjusted for the randomisation factors. A sensitivity analysis will be performed using the safety analysis set. Secondary time-to-event outcomes will be analysed using Cox proportional hazards models or competing risks analyses, as appropriate. Quality of life and functional outcomes will be analysed using longitudinal models, with joint modelling considered where appropriate. Statistical significance will be defined as p<0.05.

### Economic methods

The economic evaluation will adopt the perspective of the National Health Services and Personal Social Services operating in the UK. A resource use questionnaire that includes items relating to primary, hospital care and personal social care services will be administered during clinic visits or calls at baseline, 6 weeks, 12 weeks and 26 weeks (online supplemental file 2). If a participant’s condition prevents self-completion, caregivers will assist in completing them on the participant’s behalf where possible, ensuring the participant’s input is captured. For trial participants receiving care in England, use of hospital care services will be obtained via requests for bespoke Hospital Episode Statistics to NHS Digital England at the end of the trial. Unit costs will be obtained from the NHS reference costs,^28^ the compendium of Unit Costs of Health and Social Care and the British National Formulary.^29^ Costs will be based on the most recent available at the time of analysis.

Health outcomes will be assessed from participants’ responses to the EQ-5D-5L questionnaire, administered at baseline, 6 weeks, 12 weeks and 26 weeks. Utilities will be calculated using the National Institute for Health and Care Research preferred method at the time of analysis (currently the 5 L/3 L cross-walk tariff.^30^

Patient-level cost and utilities will be analysed using regression models, taking into account a lack of baseline utility score, to estimate mean values associated with clinically meaningful health states.^31^ These states will be the basis of a Markov model to extrapolate costs and consequences over patients’ lifetime.^32^ While the base-case economic evaluation will adopt a lifetime horizon of analysis, we will also report costs and quality-adjusted life year, and estimate the incremental cost-effectiveness ratio over the 26-week follow-up period of the trial.

A stochastic sensitivity analysis will be carried out to assess the probability of cost-effectiveness for given threshold values of willingness to pay. This will consider simultaneous uncertainty in parameter inputs and provide evidence suitable to decision makers on the cost-effectiveness of restarting DOACs at different times. Additional sensitivity and scenario analyses will be undertaken to account insofar as possible, different sources of uncertainty.

### Oversight and monitoring

The Liverpool Clinical Trials Centre (LCTC) acts as the Clinical Trials Unit and is responsible for day-to-day trial management, including data management, operation of the randomisation system, central monitoring and safety reporting on behalf of the Sponsor.^33^ LCTC is part of the University of Liverpool (https://lctc.org.uk). Trial management, monitoring and analysis functions are delivered centrally, including expedited regulatory safety reporting and operational support to participating sites. Strategic oversight is provided by an independent Trial Steering Committee (TSC), which reviews trial conduct, considers recommendations from the IDSMC, and advises the sponsor and funder on the continuation or modification of the trial. The IDSMC provides recommendations regarding trial continuation or modification, such as confirming study assumptions or proposing adjustments to the sample size.

## Ethics and dissemination

The study was approved on 14 October 2024 by the Berkshire Research Ethics Committee (reference: 24/SC/0298). Written informed consent will be obtained from all participants prior to enrolment (online supplemental file 3). Participant data will be collected and processed in accordance with the UK GDPR (General Data Protection Regulation) and the Data Protection Act 2018. Identifiable information will be stored securely and transferred separately from pseudonymised clinical data, which will be coded with a unique trial identifier. Access will be restricted to authorised members of the research team, and all data will be stored on secure servers. Substantial amendments to the protocol or participant-facing materials will be submitted to the Research Ethics Committee, Health Research Authority, Medicines and Healthcare products Regulatory Agency, and participating sites for approval prior to implementation. Approved changes will be circulated to investigators and documented through version control in the Trial Master File amendment history.

The results of the trial will be disseminated to participants, healthcare professionals, policy-makers and the wider public. Findings will be submitted for publication in peer-reviewed journals and presented at scientific conferences. Trial outcomes will also be reported in the trial registry in accordance with regulatory requirements. The trial is embedded in routine NHS practice and does not impose additional research imaging or procedures beyond standard clinical care; CT/MRI scans are limited to those performed for usual management. The end of trial is defined as database lock, after which standard site and trial closure procedures are undertaken. Participants will continue with their usual clinical care team after the trial closes.

## Discussion

The aim of this trial is to determine the optimal timing for restarting DOAC therapy following tICrH in adults who were anticoagulated prior to injury. The decision of when to restart anticoagulation represents a complex clinical dilemma that balances the competing risks of re-bleeding and thromboembolic complications. Studies suggest that early tICrH expansion (within 48 hours) on routine repeat imaging occurs in 10–37% of patients on OACs, supporting the need for temporary cessation of anticoagulation during the acute phase.^10 34^ This risk, however, reduces substantially over time.

Withholding anticoagulation for prolonged periods exposes patients to an increased risk of thrombotic complications such as stroke, myocardial infarction and venous thromboembolism.^35^ A trial of 352 patients suffering a major bleed while on a DOAC demonstrated that thrombotic events increased from approximately 4% at 1 week to 10% at 4 weeks.^36^ At 3 months, patients who had restarted anticoagulation experienced fewer strokes (HR 0.85, 95% CI 0.43 to 1.68) and deaths (HR 0.50, 95% CI 0.35 to 0.72), despite a modest increase in bleeding risk (HR 1.62, 95% CI 0.95 to 2.75) compared with those who had not restarted.^37^

The absence of high-quality randomised data in this field limits clinical confidence in current practice. Given that most affected patients are not admitted to a neurosurgical unit, but rather managed in a local out-lying hospital, clear communication of advice on restarting times can be missing altogether and lead to prolonged time off anti-coagulation and greater harm. This trial addresses the evidence gap through a multi-centre, randomised controlled design comparing 1-week versus 4-week DOAC resumption and will generate the highest level of evidence to inform future clinical practice.

### Trial status

The trial achieved trial greenlight on 25/02/2025 and the first site opened on 27/02/2025. The first patient was recruited on 10/03/2025. Recruitment is ongoing.

## Supplementary material

10.1136/bmjopen-2026-121224 online supplemental file 1

10.1136/bmjopen-2026-121224 online supplemental file 2

10.1136/bmjopen-2026-121224 online supplemental file 3
