# Supplementary material for: Timing to restart direct oral anticoagulants after traumatic intracranial haemorrhage (RESTART tICrH): study protocol for a randomised controlled multicentre trial
Source: BMJ Open. 2026 Sep 17;16(9):e121224. doi: 10.1136/bmjopen-2026-121224 (PMC13599854; doi:10.1136/bmjopen-2026-121224)
Supplement: Supplementary file 1 [file bmjopen-16-9-s001.pdf]

Study ID Number: | | | | | | | Participant initials: | | | |

RESTART tICrH

A Randomised Trial of Timing to Restart Direct Oral Anticoagulants after

Traumatic Intracranial Haemorrhage

Resource use questionnaire

Questionnaire to be completed at:

Baseline

*This questionnaire is about your use of healthcare and other services **over the past three months and including this visit.** We are asking these questions to calculate the overall costs of care.*

*For each question, please think about the healthcare you received, or any costs incurred that relate to the **Traumatic Intracranial Haemorrhage (bleed in your brain)** as well as those that relate to **any other illness**.*

Please answer all these questions, even if you have not used any services. Add 'zeros' if you did not use a healthcare resource, a box left blank cannot be interpreted as 'zero'. If you cannot remember or don't know the exact details, please give your best answer.

The first part of each question requires a "Yes" or "No" answer. For "Yes" answers, please complete all following boxes.

Date of completion (DD/MM/YYYY): __ __ / __ __ / __ __ __ __

RESTART-tICrH

Study ID Number: | | | | | | |

Participant initials: | | | |

1. In the last 3 months (including today), have you had an appointment with a doctor or nurse at a GP surgery?

Yes ☐ Please go to question 2.

No ☐ Please continue to question 3.

2. Please enter the number of times you had an appointment with a doctor or nurse at a GP surgery in the last 3 months (including today) or enter zero if no appointment took place.

	Reasons related to bleed in the brain	Other reasons
Doctor appointment at surgery		
Doctor telephone or video appointment		
Doctor home visit (call-out)		
Practice nurse appointment at the surgery		
Practice nurse telephone or video appointment		
Nurse home visit (call-out)		

Study ID Number: | | | | | | | Participant initials: | | | |

3. In the last 3 months (including today), have you received any investigation(s) and/or test(s) e.g., ultrasound, endoscopy/blood or urine test, etc.?

Yes ☐ Please go to question 4.

No ☐ Please continue to question 5.

4. Please enter the number of times below you had an investigation/test of that type in the last 3 months (including today) or enter zero if no investigation/test of that type took place.

	Reasons related to bleed in brain	Other reasons
CT scan		
Ultrasound		
MRI scan		
Endoscopy		
Chest X-Ray		
ECG		
ECHO		
Ultrasound scan dopplers		

For any other tests e.g., blood tests, urine tests, photographs, etc., please specify below

	Reasons related to bleed in the brain	Other reasons

5. In the last 3 months (including today), have you had any hospital outpatient or day patient visits?

Yes ☐ Please go to question 6.

No ☐ Please continue to question 7.

6. Please enter the number of times below you had any hospital outpatient or day patient visits in the last 3 months (including today) or enter zero if no appointment of that type took place.

		Number of times	Please specify specialist: *e.g., neurologist, cardiologist.*
Had an outpatient clinic appointment: *doctor - face to face*	Related to bleed in brain		
	Other reasons		
Had an outpatient clinic appointment: *doctor - telephone or video*	Related to bleed in brain		
	Other reasons		
Had an outpatient clinic appointment: *nurse - face to face*	Related to bleed in brain		
	Other reasons		
Had an outpatient clinic appointment: *nurse - telephone or video*	Related to bleed in brain		
	Other reasons		
Attended hospital for a day care appointment	Related to bleed in brain		
	Other reasons		

7. In the last 3 months (up to today), have you been admitted to hospital as inpatient (e.g., stayed overnight in hospital)?

Yes ☐ Please go to question 8.

No ☐ Please continue to question 9.

8. For each hospital stay in the last 3 months (up to today), please enter the duration (number of nights), reason and where you stayed in the table below, or enter zero if you did not stay in hospital.

	Hospital stay	Number of nights	Please specify reason: *e.g., heart problems, breathing problems, urine infection, heart attack, stroke, etc.*	Please specify location: *e.g., ward, intensive care unit (ICU), high dependency unit (HDU), rehabilitation unit.*
Reasons related to bleed in brain	First			
	Second			
	Third			
	Fourth			
	Fifth			
Other reasons	First			
	Second			
	Third			
	Fourth			
	Fifth			

9. In the last 3 months (including today), have you visited the accident and emergency department, or required an ambulance?

Yes ☐ Please go to question 10.

No ☐ Please continue to question 11.

10. Please enter the number of times below you visited the accident and emergency department, or required an ambulance, in the last 3 months (including today) or enter zero if no visit of that type took place.

	Reasons related to bleed in brain	Other reasons
Visited the accident and emergency department (A&E or casualty), but not in an ambulance		
Called an ambulance but was NOT taken to hospital		
Called an ambulance and was taken to accident and emergency department (A&E or casualty)		

11. In the last 3 months (including today), have you had any appointment with any other health or care professional e.g., dietician, social worker, health visitor, etc.?

Yes ☐ Please go to question 12.

No ☐ You have completed the questionnaire.

12. Please enter the number of times below you had any appointment with any other health or care professional in the last 3 months (including today) or enter zero if no appointment of that type took place.

	Reasons related to bleed in brain	Other reasons
Health visitor		
Social worker		
Physiotherapist		
Psychologist		
Occupational therapist		
Speech & language therapist		
Dietician		

For any other visits *e.g., counsellor, pharmacist, support and outreach worker, etc.,* please specify below

	Reasons related to bleed in brain	Other reasons

Thank you for taking the time to complete this questionnaire and supporting our research.
