# Supplementary material for: Timing to restart direct oral anticoagulants after traumatic intracranial haemorrhage (RESTART tICrH): study protocol for a randomised controlled multicentre trial
Source: BMJ Open. 2026 Sep 17;16(9):e121224. doi: 10.1136/bmjopen-2026-121224 (PMC13599854; doi:10.1136/bmjopen-2026-121224)
Supplement: Supplementary file 2 [file bmjopen-16-9-s002.pdf]

RESTART tICrH

<Trust/Site address 1>
<Trust/Site address 2>
<Trust/Site address 3>
<postcode>
Tel: <telephone number>

Adult Information Sheet for

RESTART tICrH: A Randomised Trial of Timing to Restart Direct Oral Anticoagulants after Traumatic Intracranial Haemorrhage (tICrH)

- You have been invited to take part in a research study. Before you decide, it is important that you understand why the research is being done and what it will involve.
- Please take time to read the following information carefully. **Part 1** tells you the purpose of the study and what will happen to you if you take part. **Part 2** gives you more detailed information about the conduct of the study.
- You can ask a member of your clinical team if there is anything that is not clear, or if you would like more information.
- If you wish you can discuss it with friends, relatives and/or get independent advice via your local Patient Advice and Liaison Service (PALS) or equivalent.
- Taking part is **voluntary**. If you don't want to take part, then you don't need to give a reason.
- In this study we want to see what the best time is to start or restart a medication called a direct oral anticoagulant (medicine that makes your blood less likely to clot) after a bleed in the head caused by a head injury (known as a 'traumatic intracranial haemorrhage').
- We will be asking participants to start or restart a direct oral anticoagulant (DOAC) after 1 week or 4 weeks.
- Patients that might be able to take part will have had a traumatic intracranial haemorrhage and be aged 18 years or above.
- The study will recruit 1084 participants from around 20 trauma networks and hospitals across the United Kingdom (UK) over a 4-year period.
- In normal clinical care patients will start/restart a DOAC between 1 and 4 weeks after head injury, so this study will not put you at any additional risk. As with any medicine there are risks of side effects. There are more details of this in this information sheet.
- You will be contacted at 6, 12 and 26 weeks after your head injury to complete some questionnaires to assess your recovery. This can be done over the telephone or online.

How to contact the study team:

If you have any questions about this study, please talk to your research team:

- Principal Investigator <<Name and Telephone number>>
- Research Nurse <<Name and Telephone number>>

Contents:

Part 1

Purpose of the study and what will happen if you take part.

Part 2

Detailed Information about the conduct of the study

PART 1: Purpose of the study and what will happen if you take part

Why are we doing the **RESTART tICrH** study?

The aim of this study is to see what the best time is to start/restart a direct oral anticoagulant (DOAC) after a traumatic intracranial haemorrhage. A traumatic intracranial haemorrhage is bleeding within the head due to trauma.

Older people falling from standing height is the most common cause of head injury resulting in admission to hospital. However, there are a range of injuries at all ages that can lead to a bleed on the brain. Up to 1 in 3 patients admitted are taking a tablet medication to reduce the risk of blood clots, known as an oral anticoagulant (OAC). An OAC can increase the likelihood of bleeding in the brain. Most patients take an OAC due to an irregular heartbeat called atrial fibrillation (which increases the risk of stroke) or because of a previous stroke or blood clot. When a scan confirms bleeding in the head, OACs are nearly always stopped, but this leaves the question of when it is safe to restart them. The risk of worsening of bleeding in the head must be balanced against the risk of stroke or blood clots.

There is very little evidence on the safest time to restart OACs, but most neurosurgeons advise restarting them 1 to 4 weeks after injury.

While your OAC is stopped, there is a higher chance of having a blood clot which could cause a stroke, heart attack or clot in your legs or lungs. There is some evidence that this clotting risk doubles between 1 and 4 weeks, which is why we want to restart the OAC as soon as is safely possible. It may be that the bleeding risk is higher earlier (1 week) than later (4 weeks) but there is no evidence around this, which is what this study aims to answer.

Every individual has different risk factors for blood clots or bleeding and these can be discussed with your clinician. It may be considered better starting your medication earlier (1 week) or later (4 weeks) depending on these risk factors. However, it is likely they will not have a clear answer for you, which is why we are inviting you to take part in the study.

The type of OAC that people normally take has changed in recent years from warfarin to newer medications called Direct Oral Anti-Coagulants (DOACs). DOACs can be easier to use compared to OACs and people taking them may not need to be monitored as closely. Only people that have previously been taking a DOAC or if their doctor decides they should be restarted on a DOAC will be able to take part in this study. This study aims to compare starting/restarting DOACs at 1 week and at 4 weeks after injury.

This study will recruit 1084 patients from across 20 trauma networks and hospitals in the UK. Participants will be involved in the study for a maximum of 6 months. The results from this study will be used to help us improve the care of patients taking a DOAC after a head injury.

Why have I been chosen?

You have been chosen to participate because you have had a head injury resulting in bleeding in the head and your doctor has recommended starting or restarting a DOAC in the next 1 to 4 weeks.

Do I have to take part?

No, taking part is voluntary. It is up to you to decide whether you want to take part.

If you decide not to take part, then you will still receive the usual treatment your hospital offers. Your doctor can provide you with more information on this.

If you decide to take part, you can also choose to stop at any time without giving a reason.

If you should lose capacity during the course of the study then it will be the choice of your next of kin or advocate whether you will continue to participate in the trial. It would be good to let them know your wishes.

The decision you make on whether to take part or not will not affect the standard of care you receive now or in the future.

What will happen to me if I take part?

If you agree to take part, you will be asked to sign a consent form. You will be given a copy of the consent form and the information sheet to keep.

Once you have signed the consent form, we will check and confirm that this study is suitable for you, and you will be asked to follow the study plan (see study timeline on page 3).

If you agree to take part, you will be asked to:

- Be involved in the study for a maximum of 6 months.
- Start/restart your DOAC when you are asked to do so by the study team.
- Be contacted at 6, 12 and 26 weeks after your injury to complete some questionnaires to assess your recovery. This can be over the telephone, video call or in person.
- Take part in an optional interview to ask about your experiences of being in the study.
- Share your head scans to help us understand what type of injury you had and build a library of scans for future research.
- Report any serious medical problems (such as blood clots or bleeding) or any readmissions to hospital, to the research team as soon as you can.

What is the optional interview?

We will interview a selection of participants, either over the telephone, videocall or in person to ask about your experience of being in the study. The interviews may be in a group with other participants or individually (you can decide). The individual interview will last 30-60 minutes and the group discussion up to 2 hours and will be recorded. This interview will take place within the first 60 days of you taking part in the study. The interview is entirely optional, and you will be asked about it when you complete the consent form.

What is the Computed Tomography (CT) scan library?

As part of standard clinical care, all patients who experience a traumatic intracranial haemorrhage will have a CT scan of their head on admission to hospital and sometimes further follow-up scans as decided by the doctor making decisions about their care. In this study we want to collect these scans by linking them electronically and securely sending them to the University of Plymouth so that we can build a library of images. This will enable us to understand more about the patterns of head injury seen in patients who take part in this study and can be very helpful for future research.

Standard clinical care scans will be collected only; no additional scans will be completed as part of the study. We will use CT scans that you have already had for your normal clinical care to confirm your eligibility and to inform the study outcomes. These CT scans will also be stored securely and anonymously at the University of Plymouth so that we can look at these scans in more detail and they can be used in the future in further research.

CT Head imaging is part of your routine care. If you take part in this study you will not undergo any additional x-ray imaging procedures. These procedures use ionising radiation to form images of your body and/or provide treatment and/or provide your doctor with other clinical information. Ionising radiation may cause cancer many years or decades after the exposure. The chances of this happening to you are the same whether you take part in this study or not.

Study Timeline

Week 0

You will be asked to sign a consent form. Your doctor or nurse will collect some information from you and your medical records, ask you to complete some questionnaires and let you know when you should start/restart your DOAC.

Week 6

You will be asked some questions about your recovery either via telephone/remote consultation or in person. You will be asked how you have been since restarting your oral anticoagulant and if you have experienced any problems.

Week 12

You will be asked to complete questionnaires about your recovery either via telephone/remote consultation or in person. You will be asked how you have been since restarting your oral anticoagulant and if you have experienced any problems.

Week 26

You will be asked to complete questionnaires about your recovery either online telephone/remote consultation or in person. You will be asked how you have been since restarting your oral anticoagulant and if you have experienced any problems.

**Procedure**	**Description**	**Research Treatment or Standard of care**
Starting/restarting DOAC	Once randomised into the study you will be told at which timepoint you should start/restart your DOAC. You will be reminded when it is time to start/restart.	***Research Treatment***
Completing study questionnaires – Modified Rankin Scale	The modified Rankin Scale (mRS) is a commonly used scale for measuring the degree of disability or dependence in daily activities. This will be completed at baseline and 26 weeks from joining the study, this may be completed earlier if you no longer wish to take part in the study.	***Research Treatment***
Completing study questionnaires – Barthel Index	The Barthel index is a scale that measures someone's ability to complete activities of daily living. This will be completed at baseline and 26 weeks from joining the study, this may be completed earlier if you no longer wish to take part in the study.	***Research Treatment***
Completing study questionnaires - Extended Glasgow Outcome Scale	The Glasgow Outcome Scale measures functional outcome. This will be completed at baseline and 26 weeks from joining the study, this may be completed earlier if you no longer wish to take part in the study.	***Research Treatment***
Completing study questionnaires - Rockwood Frailty Score	The Rockwood Frailty Score measures the level of frailty. This will be completed at baseline and 26 weeks from joining the study, this may be completed earlier if you no longer wish to take part in the study.	***Research Treatment***
Health Economics Questionnaires – EQ-5D-5L and resources use questionnaires	The EQ-5D-5L and resources use questionnaire will be used to collect information on health and whether you have used any health services whilst taking part in the study. This will be completed at baseline, 12 weeks and 26 weeks from joining the study and may be completed earlier if you no longer wish to take part in the study.	***Research Treatment***
CT Scan	As part of standard clinical care all patients who experience traumatic intracranial haemorrhage will have a CT scan and sometimes several follow-up CT scans, in order to guide management of the head injury. We want to collect images from these standard clinical care scans at baseline and up to 12 weeks for our CT repository. We will also review any standard care scans that you may have up to 26 weeks to check eligibility and inform the study outcomes.	**Standard of Care**

What is this study testing?

We want to test the timepoint after head injury at which DOAC should be started/restarted. The study is comparing starting/restarting at 1 week or 4 weeks after head injury.

How will I know which treatment I'm going to have?

In research studies we often split participants up into groups to look at how different treatments work. In the RESTART tICrH study, participants will be split into two groups at random:

- One group will start/restart at 1 week
- The other group will start/restart at 4 weeks

It is really important that each group in the RESTART tICrH study has a similar mix of participants in it, so we know that if one group of participants does better than the other it is very likely to be because of the timing of starting/restarting the DOAC and not because there are differences in the types of participants in each group.

We use a computer programme that puts participants into groups 'at random' – you might hear this described as 'randomisation' or 'random allocation', but they all mean the same thing.

In the RESTART trICH study you are equally likely to be in the group starting/restarting a DOAC at 1 week as you are to be in the group starting/restarting a DOAC at 4 weeks.

Your healthcare team will let you know which group you are in.

It is recognised that anticoagulants known as DOACS are now preferable to vitamin K antagonists (e.g., warfarin) as they are safer. If you are taking warfarin you may be asked to start a DOAC (apixaban, dabigatran, rivaroxaban or edoxaban) rather than warfarin if you decide to take part in the study.

What are the alternatives for treatment?

If you choose not to take part in this study, then the doctor looking after you will choose the best treatment for you.

What are the benefits and risks of taking part?

There is no clear evidence for the best time to start/restart a DOAC after a head injury. There is the potential for all participants in the study to benefit from taking part, because regardless of which group you are allocated to, all participants will be taking a DOAC by 4 weeks. Although there may be no direct benefit to you by taking part, we hope that the results from the study will help doctors and patients like you in the future when making decisions about treatment.

Both time-points for starting/restarting the DOAC have been shown to improve symptoms, but we do not know which time is best. In normal clinical care patients will start/restart a DOAC between 1 and 4 weeks after head injury, so this study will not put you at any additional risk.

There are four types of DOACs that are usually prescribed, you will be prescribed one that patients would usually get in your hospital. With all anticoagulants it is important to look out for signs of bleeding when taking them, please contact your Doctor at your Hospital or your General Practitioner (GP) if you notice any signs of bleeding. There are certain medications that should not be taken with a DOAC, your doctor or nurse will discuss this with you. Each type comes with adverse reactions (side effects) as with any medication and these are listed below:

Eliquis (Apixaban)

Common adverse reactions seen in other patients include haemorrhage (bleeding), contusion (bruising), epistaxis (nose bleed), and haematoma (collection of blood under the skin), anaemia (low number of red blood cells), thrombocytopenia (low platelet count), eye haemorrhage (bleeding in the eye), hypotension (low blood pressure), nausea, gastrointestinal (GI) haemorrhage (bleeding in the digestive system), haemorrhoidal haemorrhage (rectal bleeding), mouth haemorrhage (bleeding in the mouth), rectal haemorrhage (bleeding from the bottom), gingival bleeding (bleeding gums) gamma-glutamyltransferase (GGT) increased (increase in GGT which is an enzyme found throughout the body), alanine aminotransferase (ALT) increased (increase in ALT which is an enzyme

found throughout the body), skin rash, haematuria (blood in urine) and abnormal vaginal haemorrhage, urogenital haemorrhage (bleeding from female reproductive system).

Pradaxa (Dabigatran)

The most common adverse reactions seen in other patients include anaemia (low number of red blood cells), epistaxis (nose bleed), GI haemorrhage (bleeding in the digestive system), abdominal pain, diarrhoea, dyspepsia (indigestion), nausea, rectal haemorrhage (bleeding from the bottom), skin haemorrhage (bleeding into the skin) and genitourological haemorrhage, including haematuria (bleeding from female reproductive system and blood in urine).

Lixiana (Edoxaban)

The most common adverse reactions seen in other patients include epistaxis (nose bleed), haematuria (blood in urine) and anaemia (low number of red blood cells), dizziness, headaches, abdominal pain, Lower GI haemorrhage, upper GI haemorrhage (bleeding in the digestive system), oral/pharyngeal haemorrhage (bleeding from mouth and throat), nausea, blood bilirubin increased, gamma-glutamyltransferase (GGT) increased (increase in GGT which is an enzyme found throughout the body), cutaneous soft tissue haemorrhage (bleeding into the skin), rash, pruritus (itching of the skin), macroscopic haematuria/urethral haemorrhage (blood in urine), vaginal haemorrhage, puncture site haemorrhage and abnormal Liver function tests.

Xarelto (Rivaroxaban)

The most common adverse reactions seen in other patients include epistaxis (nose bleed) and GI tract haemorrhage (bleeding in the digestive system), anaemia (low number of red blood cells), dizziness, headaches, eye haemorrhage (bleeding in the eye), hypotension (low blood pressure), haematoma (bleeding under the skin), haemoptysis (coughing blood), gingival bleeding (bleeding gums), abdominal pains, dyspepsia (pain in the upper abdomen), nausea, constipation, diarrhoea, vomiting, Increase in transaminases, pruritus (itching of the skin), rash, ecchymosis (bruise), cutaneous haemorrhage subcutaneous haemorrhage (bleeding into the skin), pain in extremity, urogenital tract haemorrhage (bleeding in urinary tract), renal impairment, fever, peripheral oedema (accumulation of fluid in legs, ankles, arms or hands), decreased general strength and energy and postprocedural haemorrhage.

What happens if I change my mind?

If at any point you decide to stop taking part in the study, you will still receive treatment and the follow-up usually offered by your Hospital or Neurosurgical unit.

If you do decide to stop taking part, we will ask you if you would like to:

· continue to complete follow up visits for the study **or**

· stop taking part with no more study visits.

Information on how we will handle your information in the event of your withdrawal is detailed in Part 2 of this Information Sheet.

What if new information becomes available?

Sometimes during a research project, important new information becomes available about what is being studied. If this happens, your doctor will tell you about it and discuss with you whether you want to, or should, continue in the study. If you decide to withdraw your doctor will plan for your care to continue. If you decide to continue in the study, you will be asked to sign an updated consent form.

On receiving new information your doctor might consider it to be in your best interests to withdraw you from the study. He/she will explain the reasons and arrange for your care to continue.

If the study is stopped for any other reason, you will be told why, and your continuing care will be arranged.

What happens when the study stops?

At the end of the study, you will continue to be treated by your clinical team.

It is intended that the results of the study will be presented at conferences and published in medical journals so that we can explain to the medical

community what our research results have shown. They may also be used to apply to the regulatory authorities to make the drug widely available and/or for research related to the development of pharmaceutical products, diagnostics or medical aids. Confidentiality will be ensured at all times, and you will not be identified in any publication.

Any information derived directly or indirectly from this research, as well as any patents, diagnostic tests, drugs, or biological products developed directly or indirectly, as a result of this research, may be used for commercial purposes. You have no right to this property or to any share of the profits that may be earned directly or indirectly as a result of this research. However, in signing this form and donating scan data for this research, you do not give up any rights that you would otherwise have as a participant in research.

What if there is a problem?

Any complaint about the way you have been dealt with during the study or any possible harm you might suffer will be addressed. Detailed information is given in Part 2 of this information sheet.

Will my taking part in the study be kept confidential?

Yes. All the information about your participation in this study will be kept confidential. Detailed information on this is given in Part 2.

PART 2: Detailed Information about the conduct of the study

Who is running the study?

The Walton Centre National Health Service (NHS) Foundation Trust is the Sponsor of this study and is responsible for managing it. They are based in Liverpool in the United Kingdom. They have asked that the day to day running of the study is carried out by a team based at the Liverpool Clinical Trials Centre (LCTC, part of the University of Liverpool), researchers from the University of Plymouth (part of the central study team) and health economics researchers from Bangor University (part of the central study team).

The study has been reviewed by the Health Research Authority the National Research Ethics Service Committee, and the Medicines and Healthcare products Regulatory Agency (MHRA), to make sure that the study is scientifically and ethically acceptable.

This study is funded by the National Institute for Health and Care Research (NIHR) Health Technology Assessment programme. Award number NIHR152506.

Your doctor will not receive any personal payment for including you in this study. The hospital may receive additional funding to help with any extra costs that supporting this study might incur.

How will my information be collected and handled?

The Walton Centre NHS Foundation Trust, University of Liverpool, University of Plymouth and Bangor University are the joint Data Controllers for this study and will need to use information from your medical records for this research project.

This information will include your NHS number/ Postcode for health economic research and initials/ name/ date of birth as part of consent. Your contact details will be collected if you agree to be contacted for the optional interview or future research.

Individuals from the Walton Centre NHS Foundation Trust, the LCTC, University of Plymouth and Bangor University, NHS organisations and regulatory organisations may look at your medical and research records to check the accuracy of the research study and that it is being done properly.

People who do not need to know who you are will not be able to see your name or contact details. Your data will have a code number (Study ID) instead.

Data will be sent from your hospital or neurosurgical unit to the LCTC.

The LCTC will send data securely to Bangor University for health economics analysis. The LCTC will also send personal data (NHS Number, Name, Date of Birth and Postcode) securely to NHS England to request that information about any visits you have had to health care services or hospital admissions for the purposes of health economics analysis are provided to Bangor University (England only). The data provided by the LCTC and NHS England to Bangor University will be linked to your study ID. Bangor University will not receive any of your personal data. If you do not want us to collect this information from NHS England then please let us know.

Your hospital or neurosurgical unit will send standard care brain scan images to The University of Plymouth. These images will be sent securely using a system called the Image Exchange Portal (IEP) provided by Sectra. This service is used by almost every hospital in the UK. When your images are sent through the IEP they will contain your name, NHS number and date of birth. So that the team in The University of Plymouth can link your images to your study participant number the LCTC will need to send a copy of your consent to The University of Plymouth securely. The images will be linked to study participant number by an administrator at the University of Plymouth who is independent of the study. Once the images have been linked to you, they will be added to the University of Plymouth research portal at which time they will only contain your study participant number. This means that the people who will then look at your images will not be able to identify you. Researchers at the University of Plymouth will look at your images together with some data that we collect in this study so that we can report the characteristics of participants in the study. The LCTC will send data to The University of

Plymouth. The University of Plymouth will store your images anonymously for future research.

We will notify your GP that you will be taking part in the study for their information.

We will keep all information about you safe and secure.

Once we have finished the study, we will keep the data for 25 years, so we can check the results (if needed). We will write our reports in a way that no-one can work out that you took part in the study.

What are my choices about how my information is used?

You can stop being part of the study at any time, without giving a reason, but we will keep information about you that we have already collected. If you choose to stop taking part in the study, we would like to continue collecting information about your health from your hospital. If you do not want this to happen, tell us and we will stop.

In some cases, however, we may need to continue to collect limited information about any side-effects of the study treatment you may experience. We will only do this where we are required to do so by law.

We need to manage your records in specific ways for the research to be reliable. This means that we won't be able to let you see or change the data we hold about you.

Information sharing for other research

When you agree to take part in a research study, the information about your health and care may be beneficial to researchers running other research studies in the Walton Centre NHS Foundation Trust, the LCTC, the University of Plymouth and Bangor University and in other organisations. These organisations may be universities, NHS organisations or companies involved in health and care research in this country or abroad. Your information will only be used by organisations and researchers to conduct research in accordance with the UK Policy Framework for Health and Social Care Research, or equivalent standards**.**

If you agree to take part in this study, you will have the option to take part in future research using your data saved from this study.

Where can I find out more about how my information is used?

You can find out more about how we use your information:

- at the LCTC website: https://lctc.org.uk/
- at www.hra.nhs.uk/information-about-patients
- by asking one of the research team
- by sending an email to **<site email>,** or
- by ringing us on **<site phone number>**
- in the Health Research Authority leaflet available from www.hra.nhs.uk/patientdataandresearch
- by contacting the University of Liverpool Data Protection Officer on LegalServices@liverpool.ac.uk
- by contacting the Walton Centre NHS Foundation Trust Data Protection Officer on wcft.dpo@nhs.net
- by contacting the University of Plymouth Data Protection Officer on dpo@plymouth.ac.uk
- by contacting the Bangor University Data Protection Officer on sarah.riley@bangor.ac.uk
- In the LCTC's "Privacy Notice" available from: https://www.lctc.org.uk/privacy
- In the Walton Centre NHS Foundation Trust "Privacy Notice" available from: https://www.thewaltoncentre.nhs.uk/your-information-your-rights.htm
- In the University of Plymouth "Privacy Notice" available from: https://www.plymouth.ac.uk/research/governance/research-participant-privacy-notice
- In the Bangor University "Privacy Notice" available from: https://cheme.bangor.ac.uk/data-protection.php.en

If you are not happy with the way your information is being handled, or with the response received from us, you have the right to lodge a complaint with the Information Commissioner's Office at Wycliffe House, Water Lane, Wilmslow, SK9 5AF (www.ico.org.uk).

What if there is a problem?

If you have a concern about any aspect of this study, you should ask to speak with one of your research team who will do their best to answer your questions.

If you remain unhappy and wish to complain formally, you can do this by contacting local NHS Patient Advice

and Liaison Service (PALS) or equivalent. Members of your local hospital team should be able to provide this information to you.

Every care will be taken in the course of this clinical study. However, in the unlikely event that you are harmed by taking part in this research project, there are no special compensation arrangements. If you are harmed and this is due to someone's negligence, then you may have grounds for a legal action for compensation against the NHS Trust where you are being treated but you may have to pay for your legal costs. The normal National Health Service complaints procedures should be available to you.

Thank you for taking the time to read and consider this information sheet. Should you decide that you can take part in the study, you will be given a copy of the information sheet and a signed consent form to keep.

RESTART tICrH

<Trust/Site address 1>
<Trust/Site address 2>
<Trust/Site address 3>
<postcode>
Tel: <telephone number>

FOR SITE USE ONLY:

Site Name:	
Participant Study Number	
Participant Initials:	Participant DOB: / /

Adult Consent Form

To be completed by the participant:

Once you have read and understood each statement **please enter your initials in each box.**	Initial
1. I have read and understood the information sheet for this study. I have had the opportunity to ask questions and have had these answered satisfactorily.	
2. I understand that participation is voluntary and that I am free to withdraw from the study at any time, without giving a reason, and without my care or legal rights being affected. However, the study team may need to collect some limited information for safety reasons.	
3. I agree to take part in the above study.	
4. I give permission for a copy of this fully completed consent form to be sent to the LCTC (where it will be kept in a secure location) to allow confirmation that my consent was given.	
5. I understand that relevant sections of my medical notes and any data collected during the study may be looked at by authorised individuals from the central study team and representatives of the Sponsor, regulatory authorities and the local NHS Trust. I give permission for these individuals to have access to my records and data.	
6. I agree to my GP being informed of my participation in the study.	
7. I agree for my routine care CT scans (which will contain name, NHS number and date of birth), study data and a copy of this consent form to be transferred to the University of Plymouth and for The University of Plymouth to store CT scan images anonymously to be looked at in future research studies	
8. I agree for the relevant data on my NHS hospital admissions and treatment to be collected for the purposes of this study and understand this will include accessing electronic NHS health care records from NHS England for the financial years commencing 6 months before the start of the study and covering the duration of the study, for health economic analysis **(for England only)**.	
9. I agree for my personal data (including name, postcode, date of birth, NHS number) and a copy of this consent form to be shared with NHS England so they can provide Health Economic researchers working on the study with information regarding my medical data and hospital attendances **(for England only)**.	

NHS number:	
Postcode	

10. I understand that my data will be kept by the Data Controllers and all others archiving data and at my hospital in a confidential manner for 25 years from the end of the study.	
The statements below are **optional** (you can still take part in the study even if you do not wish to agree to these):	
11. I agree to allow information and data or results arising from this study to be used in future healthcare and/or medical research providing my confidentiality is maintained.	
12. I agree that I may be contacted to take part in the optional interviews to ask about my experiences of being in the study. (if you agree to this statement provide your details below):	

Telephone number:	
Email address:	

RESTART tICrH

<Trust/Site address 1>
<Trust/Site address 2>
<Trust/Site address 3>
<postcode>
Tel: <telephone number>

FOR SITE USE ONLY:

Site Name:														
Participant Study Number														
Participant Initials:				Participant DOB:			/			/				

Adult Consent Form

13. I agree that I may be contacted in the future in relation to this or other related studies.
(if you agree to this statement provide your details below):

Telephone number:											
Email address:											

To be completed by the participant:

Your full name (please print):			
Your signature:		Date:	

To be completed by the Researcher (after participant has completed the form):

Researcher full name (please print):			
Researcher signature:		Date:	

Please file the original wet-ink copy in the RESTART tICrH Investigator Site File, and make three copies: one for the participant, one for the medical notes and one to be sent to the LCTC.

To be completed by the Impartial Witness (only if the participant is unable to sign the consent form):

Witness full name (please print):			
Witness signature:		Date:	
Participant's full name (please print):			
