# Supplementary material for: Timing to restart direct oral anticoagulants after traumatic intracranial haemorrhage (RESTART tICrH): study protocol for a randomised controlled multicentre trial
Source: BMJ Open. 2026 Sep 17;16(9):e121224. doi: 10.1136/bmjopen-2026-121224 (PMC13599854; doi:10.1136/bmjopen-2026-121224)
Supplement: Supplementary file 3 [file bmjopen-2026-121224.reviewer_comments.pdf]

PEER REVIEW HISTORY

BMJ Open publishes all reviews undertaken for accepted manuscripts. Reviewers are asked to complete a checklist review form and are provided with free text boxes to elaborate on their assessment. These free text comments are reproduced below.

ARTICLE DETAILS

Title (Provisional)

Timing to Restart Direct Oral Anticoagulants after Traumatic Intracranial Haemorrhage (RESTART tICrH): study protocol for a randomised controlled multicentre trial

Authors

Edlmann, Ellie; Kannan, Siddarth; Wright, Laura; Hardwick, Ben; Brown, Michaela; Gornall, Matthew; McLoughlin, Terence; Haunton, Victoria; Pijeira Perez, Yankier; Hughes, Dyfrig A; Vine, Nic; Grantham, Helen; Milling, Truman J; Lane, Deirdre A; Lip, Gregory YH; Jenkinson, Michael D.; Mcmahon, Catherine J.

VERSION 1 - REVIEW

**Reviewer**	**1**
**Name**	**Holbrook, Anne**
**Affiliation**	**McMaster University**
**Date**	**09-Jun-2026**
**COI**	**None**

The protocol describes a pragmatic randomized trial protocol to compare time to restart anticoagulation after a traumatic intracranial hemorrhage event while taking an oral anticoagulant.

This is an important scientific question and the authors do reasonably articulate the clinical trade-offs involved in earlier vs later restart of anticoagulation.

The protocol provides a general description of a trial but lacks important details in many many areas as outlined in comments and concerns attached to the protocol.

However, my biggest concern lies with the effort to publish a protocol nearly 2 years after recruitment has already started. This we consider to be a misinterpretation of the peer review process. Since there will be little room to incorporate any of the suggestions, there seems to be little point to the peer review.

Further detailed comments are contained in the draft itself.

**** The reviewer provided a marked copy with additional comments. Please contact the publisher for full details. ****

**Reviewer**	**2**
**Name**	**Hosokawa, Kazuya**
**Affiliation**	**Kyushu University**
**Date**	**10-Jun-2026**
**COI**	**None**

General comment

This is a clinically important study protocol addressing a relevant unresolved question: when to restart DOAC therapy after traumatic intracranial haemorrhage. The multicentre pragmatic randomised design is appropriate and has the potential to inform clinical practice.

However, several key methodological points require clarification. In particular, the composite primary endpoint is broadly defined, the handling of missing outcome data is not prespecified, the assumptions behind the sample size calculation are not sufficiently transparent, and the rationale for selecting 1 week versus 4 weeks as the two strategies needs to be better justified.

Major comments

1. The primary endpoint should be defined more precisely.

The current definition of the haemorrhagic component is too broad and may include events of very different clinical importance. Terms such as “haematoma expansion,” “change in clinical status,” and “cessation of anticoagulation” should be operationally defined. For example, anticoagulation interruption should have a prespecified minimum duration, such as >24 hours, and should be linked to a clinically relevant bleeding concern. The authors should also consider restricting primary haemorrhagic events to clinically meaningful events, such as those requiring surgery, reversal therapy, escalation of care, hospitalisation, or clinically mandated interruption of anticoagulation. Minor radiological changes managed conservatively may be better reported as secondary or exploratory outcomes.

2. The components of the composite endpoint should be reported separately.

The composite endpoint combines haemorrhagic and thrombotic events, but these outcomes differ in mechanism, clinical severity, and expected direction of treatment effect. Earlier DOAC restart may reduce thrombotic events but increase haemorrhagic events. Therefore, the authors should prespecify separate reporting of haemorrhagic and thrombotic events, as well as the major individual components of each category, to allow clinically meaningful interpretation of the trial results.

3. Handling of missing primary outcome data should be prespecified.

The protocol states that missing data will be monitored and documented, but it does not clearly describe how missing primary outcome data will be handled in the analysis. The authors should prespecify the primary analytical approach to missing data and include appropriate sensitivity analyses, since differential loss to follow-up could affect interpretation of the 12-week binary primary endpoint.

4. The sample size assumptions require further justification.

The sample size calculation assumes a 12% composite event rate in the 4-week group and a 6% absolute reduction in the 1-week group. However, the expected rates of haemorrhagic and thrombotic events are not shown separately. Because the intervention may have opposite effects on these two components, the authors should provide the assumed event rates for haemorrhagic and thrombotic events separately over the 12-week period, together with the evidence supporting these assumptions. The clinical plausibility and importance of a 6% absolute reduction in the composite endpoint should also be explained.

5. The rationale for choosing 1 week versus 4 weeks should be clearer.

The protocol should better explain why these two specific time points were selected. In particular, the authors should clarify whether 4-week restart represents current standard care, usual practice, or a conservative comparator, and why 1-week restart was chosen as the early restart strategy. This is important for understanding the clinical relevance and generalisability of the trial.

VERSION 1 - AUTHOR RESPONSE

Reviewer 1

Page 3

Line 14- Sentence: phase III
Comment: why would this be a Phase 3 trial? There does not seem to be a relevant marketing or regulatory objective.
Response: This is a phase III trial as there is currently no regulatory guidance on the safe timing of using DOACs in this patient population. Therefore, this is using a standard treatment (DOACs) but verifying its clinical effectiveness and safety in relation to the dose timing as determined by the MHRA (https://www.gov.uk/guidance/clinical-trials-for-medicines-apply-for-authorisation-in-the-uk).

Page 4

Line 6- Sentence: most often resulted in head injury

Comment: This latter part seems very unlikely and is not consistent with previous evidence; possibly misquote? Falls are very common but head injury is not a common result of falls as a percentage.

Response: Thank-you for the comment. This data is from the UK major trauma network and therefore the patient must be classified as a major trauma (not just any fall) and does not include patients with a single fracture distal to the elbow/knee or isolated NOFs, which may explain why head injury is the most prevalent. We have clarified this in the text.

Page 4

Line 31- Sentence: clinical effectiveness of restarting

Comment: This is very vague; would add some definition. Also how is this different from the safety outcomes?

Response: The clinical effectiveness is the reduction in thrombotic events (as per the recognised clinical action of DOACs) without causing harm through haemorrhagic events – this has been clarified. The safety outcomes has been changed to reflect this is focused on the additional detail around safety (i.e. timing of the event) and overall survival.

Page 5

Line 1-2- Sentence: The primary economic objective is to estimate cost-effectiveness.
Comment: This hinges on effectiveness which needs more precise definition. Would add as a secondary objective.

Response: Although we agree that cost-effectiveness often relates to clinical effectiveness, theoretically, an intervention might show no clinical benefit but still be cheaper and thus considered cost-effective. Cost-effectiveness addresses a policy not a clinical question; hence we felt it represents a distinct primary economic outcome, conceptually different from a primary clinical outcome.

Page 5

Column: Table Outcome

Sentence: Event definition
Comment: This needs much more detail. how are events detected. How are the patients monitored? this is not something to just wait 12 weeks then look for trouble.

Patients are monitored by the clinical team using standard clinical pathways and they determine whether an event has occurred (added as footnote to table). As the trial team we ensure data is collecting at set timepoints, but any events occurring following randomisation can be reported to the trials team who are usually embedded with the clinical teams.

What is meant by change in Clinical Status? A change in conscious level, new or progressing neurological deficit (added to text)

Cessation of AC would not be part of the definition ordinarily. A patient can have a very minimal/borderline change in haemorrhage on a scan which might be part of the natural progression of the bleed, and the clinical change may be considered not to

be related, therefore we felt that the clinician deciding to stop any AC is an added measure to consolidate that the changes in imaging/clinical status are considered relevant enough to influence treatment decisions.

What are the definitions and proof for thrombotic events? how will they be adjudicated? Thrombotic events will be determined by the clinical team diagnosing any relevant event (as per table footnote added) and the routine care records leading to this diagnosis will be reviewed at the follow-up timepoints. Due to the wide range of potential thrombotic events we have not specified how this clinical diagnosis should be made (could include CTPA, Doppler ultrasound, CT or MRI head, cardiac investigations etc.) and this will not be adjudicated as long as diagnosis is made by a clinician as we are aiming to pragmatically reflect real-world clinical practice.

Since this is a composite event representing 2 extremes, did you consider a Win Ratio? We considered various options and chose a composite outcome as the most useful clinically since we felt that both ICH and VTE have equal importance to patients and their long-term recovery. We did not consider that a Win Ratio would be useful in this trial as the first event is the one that is most likely to drive the clinical decision making and the events are unlikely to be hierarchical in nature and are not graded in severity.

Death should be considered as an outcome and you will need to comment on competing risks. Death (survival) is a secondary outcome. A full statistical analysis plan will be reported prior to the trial closing and death will be a competing risk.

Response: Many thanks for the comments. The trial is open and has received positive ethics and regulatory approvals. The full protocol has more details for the site investigators and we have provided an abbreviated version here for the manuscript which is why some areas may appear to lack clarity.

Page 5

Column: Table Outcome

Sentence: Secondary outcomes
Comment: What is the justification for this large number of secondary outcomes? These will confuse the general message, so are important to have justification.

Response: The timing of events is a critically important area of understanding the final study results and its broader applications, particularly as systematic review has reported that there is very little data on the time points of events in this population, making this and any future study design very difficult.

Functional/quality of life outcomes are standard/expected outcomes in clinical research to enable us to understand the impact of the different interventions/event rates between groups on the patient's lives and longer-term day-to-day function. This is what is most important to patients. These, alongside the healthcare resource use questionnaire, are necessary to develop health economic data to support translation of this evidence into national regulation in an NHS environment.

We have added text above the table to help clarify this.

The qualitative outcomes (patient and caregiver attitudes) are discussed in more depth in the qualitative sub-study section (page 7).

Page 5

Column: Table Outcome

Sentence: Upto 1 weeks
Comment: Why are the outcomes measured at different times? If the primary outcome is measured at 12 weeks, need to justify the multiple timepoints used. Using multiple different timepoints is fairly standard in a clinical trial, e.g., for QoL we want to track changes over time to understand both the early and longer-term impact of interventions on patient health.

What is the purpose of patient and caregiver attitudes and why is this measured at different time?

Response: The patient and caregiver attitudes are part of the embedded qualitative study (page 7) as it is important to understand the impact of recommencing DOAC, especially in older frail patients. These interviews have a wide window of 60 days to complete as they are not time-critical and this allows more flexibility for patients.

Page 5

Column: Table Outcome

Sentence: Functional outcomes
Comment: Why all of these functional outcomes?
Response: Each of the chosen outcomes provides different and complementary information particularly as the main, validated head injury outcome scale used (Glasgow Outcome Scale) has been shown to potentially be less relevant to older people, therefore we wanted to also include other non-head-injury specific scales (Barthel Index and modified Rankin scale). The frailty index is a key NHS metric in assessing older people and frequently assessed as part of standard care in this population.

Page 5

Column: Table Outcome

Sentence: Healthcare resource use
Comment: Healthcare resource use is to inform economics but needs more detail. What is the perspective used - societal or healthcare plan or other? Exactly which costs will be considered? We would include the Health Resource questionnaire as appendix.
Response: The perspective has now been mentioned explicitly in the relevant section. This defines which costs are considered, and these too are listed in the "Economic methods" section.

Page 6

Line 13- Sentence: decision to use a composite primary endpoint. All patients were happy to be randomised to restarting a DOAC

Comment: This patient and caregiver involvement should be described more scientifically - how was this done? how many involved? Is this published work? Consider using the major works published on patient preferences - for example analysis of > 1000 patients in PEARL - AF in JACC Advances.
Response: As part of the trial development, we undertook PPIE activity. This is a standard approach to NIHR funded trials. The activity involved meeting patients with lived experience of taking a DOAC, to discuss the trial idea, acceptability of the primary outcome etc. This work is not published (and is not usually published) but follows the NIHR PPIE guidance. https://www.nihr.ac.uk/research-funding/application-support/working-with-people-and-communities

Page 6

Line 16- Sentence: where the patients lack capacity to consent.
Comment: How will capacity to consent be measured? there are published questionnaires for this. What is a consultee? Is this Power of attorney?
Response: Capacity is assessed by the clinical team using standard clinical assessments in place at each site. We did not stipulate how to assess capacity within the protocol as this is standard clinical care. In patients deemed not to have capacity the patient's legal representative is approached (this is the consultee and we have changed wording for consistency) to consent on the patient's behalf. In UK health research a legal representative is someone who has a close, personal relationship with the patient, they do not officially have to have power of attorney but are usually the registered next-of-kin.

Page 6

Line 19- Sentence: materials, and interpretation of findings.
Comment: What is a PPI? If you are going to emphasize the role of 2 patient /public reps, then need to describe trial governance - is there an exec committee, a DSMB, etc.?
Response: PPI. Patient and Public Involvement (see earlier response). For NIHR funded trials it is part of the standard framework to have public members on the trial management group and trial steering committee. The governance structures apply to all TMG and TSC members.

Page 6

Line 33- Sentence: The study is a Type A CTIMP: no higher than that of standard medical care.
Comment: Explain all acronyms. This is not an international standard; needs explanation
Response: CTIMP (Clinical Trial of Investigational Medicinal Product). Added to manuscript.

Page 6

Line 34- Sentence: Participants will be identified and recruited across at least 20 trauma networks in the United Kingdom.

Comment: Where are sites named?
Response: We have not named the sites in this manuscript. Open sites are listed on the trial website: https://restart-trial.org.uk

Page 6

Line 49- Sentence: 1 week who were taking an OAC prior to admission.
Comment: would be more specific - does this mean taking OAC at any time before admission or immediately before? What is the definition of a 'traumatic' ICH?
Response: We have been deliberately non-specific about the timing of the OAC prior to admission – as long as they are taking it as part of their regular medication it does not matter when the last dose was (even if it was a week ago) – as the only relevant aspect is that they should be on it and are intended to restart it, this is clarified in text. Traumatic is defined as ICH due to trauma e.g., fall and head injury rather than a "spontaneous" ICH (or stroke). These are standard terms in this population.

Page 7

Line 3- Sentence: Pregnant or nursing female.
Comment: suggest 'breast-feeding'
Response: Thanks changed as suggested

Page 7

Line 28- Sentence: prescribed as part of usual care according to the patient's prior prescription or local hospital policy.
Comment: Seems that this approach, which is more of an 'intent' to restart OAC at 1 vs 4 weeks, will disadvantage the 4 week group as there does not appear to be anyone responsible for prescribing. AT 1 week, the patient might still be in hospital, at 4 weeks patient will most likely be home and less supervised.
Response: Patients and the medical team are informed of the specific data to restart DOAC on randomisation and this is usually transcribed onto their discharge paperwork if discharged before the restart time (this is shared with the patient's GP). Patients who have been discharged are contacted directly to remind them to restart the DOAC and usually have medication already available or can get this from the GP is needed. Next of kin carers and other healthcare staff are also informed to support this process. We have not encountered any issues with this approach during the trial and patients have restarted on the correct date.

Page 8

Line 37-42- Sentence: The calculation assumed a 12% event rate (proportion of patients with haemorrhagic or thrombotic event within 12 weeks of tIcH) in the 4-week restart group and aimed to detect a 6% absolute reduction in the 1-week restart group, using a group sequential design with a single interim analysis after 50% of patients have reached 12 week follow-up or experienced an event. Sample size calculations were undertaken in SAS

Comment: The assumed event rate has to be based on data - needs citation and more rigorous justification. The anticipated absolute risk reduction is very large and not clinically believable. What data was this based on?
Response: We have added a summary on this in the sample size section. Due to the lack of data in the tICrH population alone, event rate data was calculated from a post-analysis of a trial assessing patients with a major bleed whilst on a DOAC (64% of whom had an intracranial haemorrhage) (**Milling**). The reported re-bleeding rate was around 4%, thrombotic event rate 9.7%, and composite event rate (including death) of 13.6% at 30 days. Just under 1/3 of patients had re-started their anti-thrombotic by 30 days and no thrombotic events occurred after re-starting DOACs. When using a landmark analysis to compared patients who re-started their DOAC versus not restarted, a 14-day cut-off had a HR of 0.384 for composite events. This suggests a greater than 50% reduction in events if restarting by 2 weeks, and haemorrhagic events they were similar between those who had restarted (4%) and not (4.4%). We have modestly extrapolate this data to our cohort with a combined event rate of 12% at 4 weeks and a 50% reduction in the early time point (1 week) assuming a significant reduction of thrombotic events without an increase in haemorrhagic events and is in-keeping with the event rates observed in the DOAC-trial at 1 week (**Milling**).

Page 9

Line 11-12 Sentence: No blinding will be implemented for participants, treating clinicians, site research staff, and outcome assessors.
Comment: Lack of blinding will lead to bias, less problematic given fairly discrete outcome events. However, given that the only difference between groups is the timing of restarting OAC, there does not seem to be any reason that outcome collectors could not be blinded as well as statisticians and anyone else forming results from the data.
Response: The trial was designed pragmatically as outcome measures are collected by local sites who will have been involved in patient recruitment and randomisation so not possible to blind. There will be a blinded statisticians undertaking the analysis.

Page 9

Line 32- Sentence: (Figure 1)
Comment: Fig 1 shows first follow-up at 6 weeks. This means 5 weeks after Wk1 group has re/started DOAC and only 2 weeks after Wk4 group has re/started DOAC. Would recommend comment on potential bias related to this differential in time to follow-up.
Response: The primary outcome will be measured at 12 weeks and this will inform the trial outcome. The 6 week assessment provide a timepoint to capture events rather than just collecting data at 12 weeks. This will not introduce bias for assessing the primary outcome. The starting point (randomisation following tICrH) and follow-up time is the same for all participants. The clinical question is about the strategy of restarting DOAC earlier vs. later, observed differences reflect the timing of restarting.

Page 9

Line 42- Sentence: the trial database.
Comment: There is a lack of detail on outcomes. Recommend a detailed table of outcomes with timing, what is being measured, and exactly how it will be measured.
Response: Outcomes and timing of collection are detailed in table 1 and includes the functional and QoL outcomes. We have added an appendix detailing the functional outcomes scales. EQ-5D-5L is a standard scale and we have not added this.

Page 11

Line 28- Sentence: or once an event has been observed,
Comment: please clarify what this part means
Response: This means that that primary outcome has been observed – clarified in the manuscript

Page 11

Line 21- Sentence: The primary outcome will be analysed using logistic regression
Comment: would reinterate primary outcome here again
Response: Thank-you, this is added as suggested

Page 11

Line 19-20 Sentence: A resource use questionnaire that includes items relating to primary, secondary, community and personal social care services, will be administered during clinic visits or calls at baseline, 6-, 12- and 26-weeks.
Comment: The resource use questionnaire should be an appendix and the items outlined here in text. Also would avoid country-specific jargon where possible, eg, what does 'secondary care' include?
Perspective of the economic analysis is missing.
Response: The resource use questionnaire is now included in the Appendix We have re-phrased "secondary care" to "hospital care" We have also mentioned the perspective of the evaluation.

Page 13

Line 12-14 Sentence: he Liverpool Clinical Trials Centre (LCTC) acts as the Clinical Trials Unit and is responsible for day-to-day
trial management, including data management
Comment: Would add a bit of detail for those not familiar - is this a privately-funded or public-funded unit? What are the conflicts of interest? Is this a very experience group or not? There should at least be a citation here for this group.
Response: Website citation added. LCTC part of University of Liverpool. Large experience of running clinical trial. Funded by University of Liverpool and grant income. Conflicts of interest are presented for each co-author as per publication guidelines. We have not declared conflicts of interest for the organisation.

Page 12

Line 31-32 Sentence: The study was approved on 14 October 2024 by the Berkshire Research Ethics Committee (reference:
24/SC/0298).
Comment: If REB approval is 1.5 years ago, why is publishing the protocol so delayed?
Response: We have focused efforts on opening sites for the trial and now we are recruiting steadily have elected to publish the protocol in its final format following some minor amendments made in the early stages of the trial to support recruitment.

Page 12

Line 50- Sentence: The trial is embedded in routine NHS practice and does not impose additional imaging
Comment: This is not strictly true. Indirectly the 1-week restart group is going to make clinicians nervous and there will be a lower threshold for repeating CTs and MRIs.
Similarly, from the point-of-view of a clinician who balances thrombosis-hemorrhage risk at 2 weeks post-event on average, the delay to 4 weeks will prompt more vigilance perhaps more imaging re thromboses....
Response: We have clarified that no additional research imaging is required for the trial. Other imaging is performed at the discretion of the local clinical teams. In our experience with the first >100 patients, additional imaging and vigilance is not being undertaken unless patients are unwell, as the majority of these patients are discharged home early and are well in the community. They are also not routinely followed up by the neurosurgical team in standard NHS practice, therefore often there is very little oversight of what happens to them post-discharge.

Page 13

Line 13-20 Sentence: One recent trial assessing patients with major bleeding on anticoagulation reported re-bleeding rates of 4.3% within 30 days when excluding the first three days after the initial bleed [29]. Importantly, the bleeding rate was only 4.5% among those who restarted anticoagulation within two weeks compared to those who did not restart
within the same timeframe. Another study focusing specifically on tICrH observed a 5% readmission rate for re-bleeding within 30 days in patients who did not restart their DOAC [30].
Comment: This discussion of relevant trial evidence should be introduced in the Background and Rationale.
Response: Thank-you, this is amended as suggested

Page 13

Line 47-48 Sentence: The trial achieved trial greenlight on 25/02/2025 and the first site opened on 27/02/2025. The first patient was
recruited on 10/03/2025. Recruitment is ongoing.

Comment: what is the point of requesting peer review when one knows that comments recommending change in the protocol will be received, if recruitment has already started??
Response: We are following good research practice by publishing the protocol and adherence to the SPIRIT guidelines. As per the BMJ Open guideline to peer-review of trial protocols that already have ethics / regulatory approval. It is still possible to make amendments to a trial should there be significant concerns, but in general very difficult to change a trial protocol regardless once trial funding and ethics is agreed which is prior to protocol publication anyway.

Reviewer 2

1. The primary endpoint should be defined more precisely.

Comment: The current definition of the haemorrhagic component is too broad and may include events of very different clinical importance. Terms such as "haematoma expansion," "change in clinical status," and "cessation of anticoagulation" should be operationally defined. For example, anticoagulation interruption should have a prespecified minimum duration, such as >24 hours, and should be linked to a clinically relevant bleeding concern. The authors should also consider restricting primary haemorrhagic events to clinically meaningful events, such as those requiring surgery, reversal therapy, escalation of care, hospitalisation, or clinically mandated interruption of anticoagulation. Minor radiological changes managed conservatively may be better reported as secondary or exploratory outcomes.

Response: This was considered at length and the study team felt that by fulfilling all criteria of bleeding on imaging, clinical change and stopping anti-coagulation for any time period would likely reflect a significant event. We have clarified the definition of change in clinical status in the protocol as 'altered conscious level, new or progressing neurological deficit. We have not defined the other components as the aim was to keep the trial pragmatic and truly reflective of real clinical practice. For example, if the treating clinician considers haematoma expansion is clinically relevant then they will meet the primary outcome. We are planning a sub-study analysis of imaging and therefore will have some quantifiable measure for patients with and without events.

2. The components of the composite endpoint should be reported separately.

The composite endpoint combines haemorrhagic and thrombotic events, but these outcomes differ in mechanism, clinical severity, and expected direction of treatment effect. Earlier DOAC restart may reduce thrombotic events but increase haemorrhagic events. Therefore, the authors should prespecify separate reporting of haemorrhagic and thrombotic events, as well as the major individual components of each category, to allow clinically meaningful interpretation of the trial results.

Response: The trial was designed as a pragmatic study to reflect clinical practice and the need to balance haemorrhage and thrombotic events – we considered these equally important (as did patients) hence the composite outcome. In the secondary outcomes we have separated out the haemorrhagic

and thrombotic events including time -to-event, which will provide additional information to inform future clinical practice.

3. Handling of missing primary outcome data should be prespecified.

The protocol states that missing data will be monitored and documented, but it does not clearly describe how missing primary outcome data will be handled in the analysis. The authors should prespecify the primary analytical approach to missing data and include appropriate sensitivity analyses, since differential loss to follow-up could affect interpretation of the 12-week binary primary endpoint.

Response: A full statistical analysis plan will be developed prior to data analysis. This will include how to handle missing data and sensitivity analyses.

4. The sample size assumptions require further justification.

The sample size calculation assumes a 12% composite event rate in the 4-week group and a 6% absolute reduction in the 1-week group. However, the expected rates of haemorrhagic and thrombotic events are not shown separately. Because the intervention may have opposite effects on these two components, the authors should provide the assumed event rates for haemorrhagic and thrombotic events separately over the 12-week period, together with the evidence supporting these assumptions. The clinical plausibility and importance of a 6% absolute reduction in the composite endpoint should also be explained.

Response: The clinical plausibility in relation to previous research has been added to the protocol including individual event rates.

5. The rationale for choosing 1 week versus 4 weeks should be clearer.

The protocol should better explain why these two specific time points were selected. In particular, the authors should clarify whether 4-week restart represents current standard care, usual practice, or a conservative comparator, and why 1-week restart was chosen as the early restart strategy. This is important for understanding the clinical relevance and generalisability of the trial.

Response: Thank-you, we agree this needs clarification. It was based on a UK survey of practice and review of literature with open discussion at a national meeting for consensus, this has been added to the introduction

---

VERSION 2 - REVIEW

**Reviewer**	**1**
**Name**	**Holbrook, Anne**
**Affiliation**	**McMaster University**

Date 09-Jul-2026

COI

Detailed ratings not repeated above.

The manuscript is now improved but I continue to have reservations regarding the lack of definitions, the lack of detail in several areas, most importantly for a detailed analysis plan. The authors refer to a detailed protocol, why not cite this protocol and make it available?

I find it very odd that sites seem not yet to be known despite the trial running for months - explanation would be helpful.

However, my main concern still is the request for external peer review after the trial has already started for sometime. This makes suggestions for meaningful improvement very difficult to implement.

**Reviewer**	**2**
**Name**	**Hosokawa, Kazuya**
**Affiliation**	**Kyushu University**
**Date**	**10-Jul-2026**
**COI**	

Reviewer 1 and Reviewer 2 independently converge on the same underlying concern: the composite primary endpoint contains a component — cessation of anticoagulation — that is a clinician's discretionary action rather than an objective clinical event, assessed without blinding and without independent adjudication. In an open-label trial where clinicians are aware of allocation, this creates a plausible mechanism for differential ascertainment between arms even if the underlying disease process is identical. This is very likely to be the deciding issue for this round of review. Introducing a lightweight, blinded (to allocation) adjudication step for the haemorrhagic component, together with a minimum-duration threshold for AC cessation, would directly resolve both reviewers' concerns with a proportionate amount of additional infrastructure.

VERSION 2 - AUTHOR RESPONSE

Reviewer 1

Comment: The manuscript is now improved but I continue to have reservations regarding the lack of definitions, the lack of detail in several areas, most importantly for a detailed analysis plan. The authors refer to a detailed protocol, why not cite this protocol and make it available?

Response: The detailed protocol is available in the latest version on our website but we have also added current version as an appendix. The statistical analysis plan is

being published as a separate document, as is our standard practice, to enable a comprehensive review of the statistics. Please let us know which specific definitions you feel are still out-standing and we can add these if possible. We have added some text to the event reporting section to give a more detailed explanation of this to help with understanding of the study outcomes.

"Thrombotic events are determined by imaging initiated by clinicians on the basis of patient symptoms and clinical suspicion as part of routine care, rather than any pre-detemined screening. As these are outcome events they will be screened for at all follow-up points with the research team but do not require immediate reporting. Haemorrhagic events are also determined by imaging initiated by clinicians, however, to avoid over-reporting of minor or insignificant imaging changes, must also be accompanied by a clear clinical concern with new neurological symptoms or altered conscious level. In the case of patients who have restarted their DOAC this must also be considered significant enough that the clinician wishes to stop the DOAC. There is no central adjudication or pre-specified determinants of haemorrhagic events and the study relies on pragmatic clinician-led decision making."

Comment: I find it very odd that sites seem not yet to be known despite the trial running for months - explanation would be helpful.

Response: We have a full list of sites available on the trial website, although this changes regularly as more sites continue to open and therefore is not fixed.

Comment: However, my main concern still is the request for external peer review after the trial has already started for sometime. This makes suggestions for meaningful improvement very difficult to implement.

Response: We appreciate that this appears a conflict, although it is common that protocols are not published until the study has received national funding and ethical approval through peer-review, beyond which point it is very difficult to make any meaningful changes to the core study design. We did not want to delay recruitment and therefore have started but can still make amendments to the study on advice of reviewers, if this is felt to add to the scientific value to the study.

Reviewer 2

Comment: Reviewer 1 and Reviewer 2 independently converge on the same underlying concern: the composite primary endpoint contains a component — cessation of anticoagulation — that is a clinician's discretionary action rather than an objective clinical event, assessed without blinding and without independent adjudication. In an open-label trial where clinicians are aware of allocation, this creates a plausible mechanism for differential ascertainment between arms even if the underlying disease process is identical. This is very likely to be the deciding issue for this round of review. Introducing a lightweight, blinded (to allocation) adjudication step for the haemorrhagic component, together with a minimum-

duration threshold for AC cessation, would directly resolve both reviewers' concerns with a proportionate amount of additional infrastructure.

Response: Many thanks for the comment. The study was designed as a pragmatic trial to address an area of clinical uncertainty regarding the timing of restarting DOAC in older patients with traumatic intracranial haemorrhage. The composite primary endpoint is the proportion of patients with critical haemorrhagic or thrombotic events within 12 weeks following traumatic intracranial haemorrhage (tICrH).

This judgement is made by the treating medical team, for example an expansion of the intracranial haemorrhage on CT that leads to neurological worsening, or a new thrombotic event (PE, DVT, embolic stroke) diagnosed on medical imaging. We deliberately left the haemorrhagic events to clinician discretion, rather than a centrally-adjudicated threshold, as we wanted to reflect real-world practice. As there is currently no clear evidence or guidelines on the volume or distribution of tICrH that determines whether or not a DOAC is safe to continue/restart, and this is the exact question we are seeking to answer, we did not feel it appropriate to determine a minimum AC cessation threshold. Instead, we feel that the combination of 3 factors adequately covers a real-world scenario of a “clinically relevant haemorrhagic event” – this is predominately due to the fact that it has enlarged on imaging and is causing neurological symptoms, with the natural sequalae that this should result in cessation/continued withholding of AC.

In an ideal world we would validate all imaging data for haemorrhagic events to better understand the radiological features of any re-haemorrhage. However, there are two limitations; the first is that not all sites have agreed to share raw imaging data on included patients and the second that even in sites who are sharing this data, it is not live and takes time to transfer and any adjudication of events to indicate a need for cessation would need to happen in relative “real-time”. Although broad metrics on haemorrhagic volume changes or limits could be applied by local clinicians, again this would be arbitrary given the lack of evidenced data in this field and we felt the most useful benchmark was the experience and judgement of the clinician in considering any haemorrhage as significant enough to cause symptoms and stop the AC. We will be collecting and retrospectively analysing all imaging data available, and therefore where possible can report on radiological aspects of haemorrhagic events at the end of the study for context.

We have added a section in the study harms and outcomes to further clarify this and support understanding of the study outcomes.

“Thrombotic events are determined by imaging initiated by clinicians on the basis of patient symptoms and clinical suspicion as part of routine care, rather than any pre-detemined screening. As these are outcome events they will be screened for at all follow-up points with the research team but do not require immediate reporting. Haemorrhagic events are also determined by imaging initiated by clinicians, however, to avoid over-reporting of minor or insignificant imaging changes, must also be accompanied by a clear clinical concern with new neurological symptoms or altered conscious level. In the case of patients who have restarted their DOAC this must also be

considered significant enough that the clinician wishes to stop the DOAC. There is no central adjudication or pre-specified determinants of haemorrhagic events and the study relies on pragmatic clinician-led decision making."

VERSION 3 - REVIEW

**Reviewer**	**2**
**Name**	**Hosokawa, Kazuya**
**Affiliation**	**Kyushu University**
**Date**	**09-Aug-2026**
**COI**	

Thank you for your response and for updating the manuscript with a detailed description of the event definitions and reporting criteria. I appreciate the clarification on how the primary endpoints are defined and determined within the context of this pragmatic trial. My concerns regarding the endpoint definitions have been addressed satisfactorily, and I have no further comments.
