# Supplementary material for: Timing to restart direct oral anticoagulants after traumatic intracranial haemorrhage (RESTART tICrH): study protocol for a randomised controlled multicentre trial
Source: BMJ Open. 2026 Sep 17;16(9):e121224. doi: 10.1136/bmjopen-2026-121224 (PMC13599854; doi:10.1136/bmjopen-2026-121224)
Supplement: Supplementary file 4 [file bmjopen-16-9-s003.docx]

Supplementary table 1

| Simplified Modified Rankin Scale (smRS): | The Simplified Modified Rankin Scale (smRS) is a validated measure of global disability following neurological injury and is widely used in stroke and traumatic brain injury research. It classifies patients into seven categories ranging from 0 (no symptoms) to 6 (death), with higher scores indicating greater disability. The simplified version employs a structured interview format that improves inter-rater reliability while maintaining strong agreement with the conventional modified Rankin Scale. In this study, the smRS will be used to assess overall functional disability and independence at 12 and 26 weeks following traumatic intracranial haemorrhage. |
| --- | --- |
| Barthel Index: | The Barthel Index (BI) is a validated measure of functional independence that evaluates a patient's ability to perform ten basic activities of daily living, including feeding, bathing, dressing, toileting, transfers, mobility, and stair climbing. Scores range from 0 to 100, with higher scores representing greater independence. The Barthel Index is sensitive to changes in physical function over time and has been extensively validated in stroke, traumatic brain injury, and older adult populations. It will be used to quantify recovery of functional independence during follow-up. |
| Extended Glasgow Outcome Scale: | The Extended Glasgow Outcome Scale (GOSE) is an eight-level ordinal scale developed to assess overall recovery following traumatic brain injury. It expands the original Glasgow Outcome Scale by subdividing categories of severe disability, moderate disability, and good recovery, thereby improving sensitivity to clinically meaningful differences in outcome. Scores range from 1 (death) to 8 (upper good recovery), with higher scores indicating better neurological recovery. The GOSE is one of the most widely recommended outcome measures in traumatic brain injury clinical trials and will be used to evaluate long-term neurological recovery in this study. |
| Rockwood Frailty Score. | The Rockwood Clinical Frailty Scale (CFS) is a validated nine-point clinical measure of frailty that assesses an individual's physiological reserve and vulnerability to adverse health outcomes. The scale incorporates mobility, physical activity, comorbidity burden, cognition, and dependence in activities of daily living to classify patients from 1 (very fit) to 9 (terminally ill). Frailty has consistently been shown to predict mortality, complications, functional decline, and healthcare utilisation following trauma and neurosurgical conditions, particularly in older adults. In this study, the Rockwood CFS will be used to characterise baseline frailty and to facilitate stratification and interpretation of functional outcomes. |
